# Long‐Term Stable Neural Interfaces with Nanoporous Graphene Electrodes and Hybrid Polyimide‐Aluminium Oxide Encapsulation

**DOI:** 10.1002/smtd.202501720

**Published:** 2025-11-10

**Authors:** Georgios Alexandros Katirtsidis, Xavier Illa, Nicola Ria, Elena del Corro, Eduard Masvidal Codina, Jose A. Garrido

**Affiliations:** ^1^ Catalan Institute of Nanoscience and Nanotechnology (ICN2) CSIC and The Barcelona Institute of Technology, Campus UAB Bellaterra 08193 Spain; ^2^ Instituto de Microelectronica de Barcelona IMB‐CNM (CSIC) Campus UAB Bellaterra 08193 Spain; ^3^ Biomedical Research Networking Center in Bioengineering Biomaterials, Nanomedicine (CIBER‐BBN) Barcelona 28029 Spain; ^4^ Spanish National Research Council (CSIC) Madrid 28006 Spain; ^5^ Institute of Neurosciences Universitat Autònoma de Barcelona Cerdanyola del Vallès 08193 Spain; ^6^ ICREA Barcelona 08010 Spain

**Keywords:** atomic layer deposition, flexible electronics, graphene neural interfaces, hybrid encapsulation, long‐term stability, minimally invasive implants, nanoporous microelectrodes

## Abstract

Graphene‐based thin film technology holds great promise for next‐generation neural interfaces due to graphene´s electrical, electrochemical, and mechanical properties. However, the long‐term reliability of this technology remains a challenge, primarily due to the instability of thin‐film encapsulation in physiological environments. While standard ceramic encapsulation is robust, it is not compatible with the miniaturization required for minimally invasive implants. This study demonstrates the successful integration of a hybrid encapsulation strategy, combining polyimide with atomic layer deposited (ALD) Al_2_O_3_, with nanoporous reduced graphene oxide (rGO) microelectrodes, resulting in chronically stable graphene‐based neural interfaces. The encapsulation robustness is validated using flexible interdigitated electrodes (IDEs) subjected to accelerated aging and continuous electrical stress. IDEs demonstrated stable performance after soaking for over 1.5 years in phosphate buffer saline (PBS) at 57 °C. Nanoporous graphene microelectrodes combined with the proposed encapsulation retained their structural integrity and electrochemical performance after soaking for 377 days in PBS at 57 °C, withstanding 1 billion biphasic pulses at very high charge density (1 mC cm^−^^2^) and hundreds of bending cycles without noticeable performance deterioration. This work establishes a long‐term stable graphene‐based neural interface combining nanoporous rGO electrodes and a hybrid polyimide/Al_2_O_3_ encapsulation, demonstrating a key technology advancement for the use of graphene neural interfaces in chronic brain monitoring and neuromodulation applications.

## Graphical abstract

Flexible thin‐film neural microelectrodes combining nanoporous reduced graphene oxide and a PI/Al_2_O_3_ hybrid encapsulation maintained stable electrochemical performance after soaking in PBS at 57 °C for over 377 days while they sustained the injection of 1 billion biphasic current pulses at high charge density (1 mC cm^−2^). Electrochemical characterization, Raman spectroscopy, and optical inspection validated the robustness of our neurotechnology platform in a physiologically relevant environment.

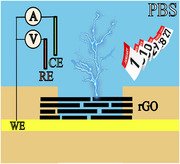

## 1 Introduction

Neural interfaces have emerged as promising therapeutic tools for treating neurological disorders and restoring impaired sensory and motor functions.^[^ ^1^ ^]^ Recent advances have focused on thin‐film implantable devices that are smaller,^[^ ^2^ ^]^ flexible,^[^ ^2^, ^3^, ^4^, ^5^ ^]^ conformable,^[^ ^2^, ^6^ ^]^ and even stretchable.^[^ ^6^ ^]^ These next‐generation neurotechnologies enable the development of microelectrode arrays capable of both high‐fidelity neural recording and high‐precision neuromodulation,^[^ ^3^, ^7^, ^8^ ^]^ laying the groundwork for increasingly sophisticated brain‐computer interfaces.

Neuromodulation applications demand stable charge injection over years of implantation, which imposes stringent requirements on the reliability of integrated material systems. In vivo, implanted electronics are subjected to multiple degradation mechanisms, including corrosion, delamination, swelling, moisture ingress, and mechanical or electrical stress,^[^ ^9^, ^10^, ^11^ ^]^ all of which can compromise performance and ultimately lead to device failure. Polymers like polyimide (PI),^[^ ^3^, ^4^ ^]^ polydimethylsiloxane (PDMS),^[^ ^6^, ^12^ ^]^ Parylene C,^[^ ^13^ ^]^ and SU‐8^[^ ^2^, ^4^ ^]^ are commonly used for encapsulation in thin‐film bioelectronics due to their flexibility and biocompatibility. However, their relatively high water vapor transmission rates (WVTR) can lead to gradual moisture penetration and material degradation under humid conditions. For instance, PDMS readily absorbs moisture and swells,^[^ ^14^ ^]^ which can compromise mechanical integrity and interfacial stability. Similarly, Parylene C films may develop microcracks that accelerate water ingress over time.^[^ ^15^, ^16^ ^]^ In addition, the properties of SU‐8 are known to deteriorate after prolonged exposure to humid environments,^[^ ^17^, ^18^ ^]^ while PI often exhibits weak interfacial adhesion, potentially leading to premature delamination in aqueous conditions.^[^ ^19^ ^]^ To address these limitations, hybrid organic–inorganic encapsulation schemes have been proposed, combining polymers with inorganic layers such as Al_2_O_3_,^[^ ^20^, ^21^, ^22^ ^]^ HfO_2_,^[^ ^20^, ^23^, ^24^ ^]^ SiO_2_,^[^ ^23^, ^25^ ^]^ and SiN_x_.^[^ ^25^ ^]^ These multilayer stacks show excellent barrier performance in accelerated aging and in vitro soaking tests, yet their durability under long‐term electrical stimulation remains insufficiently explored.

Within these approaches, PI has proven particularly advantageous due to its thermal stability, chemical resistance, and mechanical flexibility, which support compatibility with advanced microfabrication processes.^[^ ^3^, ^4^, ^7^, ^8^, ^10^, ^20^, ^21^, ^26^ ^]^ When combined with ALD‐deposited metal oxides such as Al_2_O_3_, TiO_2_, and HfO_2_, PI‐based hybrid encapsulation has demonstrated years‐long stability in physiological environments^[^ ^20^, ^21^ ^]^ (see Table S1, Supporting Information), highlighting its promise for realizing flexible and durable neural interfaces.

Meanwhile, graphene‐based thin‐film technology has emerged as a promising platform for neural interfacing applications, thanks to graphene's high electrical conductivity, mechanical flexibility, and large electrochemical potential window.^[^ ^27^ ^]^ In particular, nanoporous rGOmicroelectrodes combine scalable fabrication with an enhanced electrochemical surface area, yielding low impedance and high charge injection limits, which are critical parameters for both high‐fidelity recording and safe and effective stimulation.^[^ ^3^, ^8^, ^28^, ^29^ ^]^ Despite promising in vivo validation of flexible graphene neural interfaces and recent efforts toward clinical translation,^[^ ^30^ ^]^ no prior work has integrated graphene electrodes with a robust hybrid encapsulation optimized for chronic, active neuromodulation.

Although hybrid polymer‐oxide encapsulations have proven effective for conventional devices like interdigitated electrodes or metal tracks/planes,^[^ ^20^, ^21^, ^22^, ^24^ ^]^ their integration with graphene‐based materials has remained underexplored. The chemical inertness of graphene limits interfacial adhesion with many inorganic layers and metals, potentially leading to faster degradation. Differences in thermal budget and process compatibility can further complicate layer uniformity and mechanical stability. Furthermore, most hybrid encapsulations have been tested under accelerated aging conditions in passive mode or under continuous DC bias, with limited assessment under high‐frequency electrical stress.

Here, we bridge this gap by presenting a flexible neural interface that combines nanoporous rGO microelectrodes with a hybrid bilayer encapsulation of polyimide and ALD‐Al_2_O_3_. Polyimide contributes biocompatibility, chemical resistance, and mechanical flexibility,^[^ ^34^, ^35^ ^]^ while the ultrathin Al_2_O_3_ provides outstanding moisture barrier properties.^[^ ^9^, ^21^ ^]^ We first validated the encapsulation strategy on interdigitated test structures under accelerated aging conditions (57 °C in PBS) and electrical stress, monitoring impedance and leakage current over time. Next, we integrate the hybrid encapsulation in the fabrication of flexible arrays of rGO microelectrodes, and validate their electrochemical performance and stability during long‐term soaking, hundreds of bending cycles, and continuous billion‐pulses stimulation. Optical inspection and Raman spectroscopy confirm the structural integrity of the microelectrodes throughout the in vitro functional tests.

## 2 Experimental Section

### 2.1 Device Design and Fabrication

**Figure 1:**
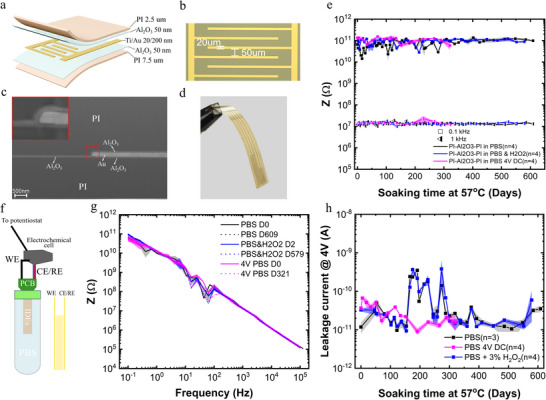
Assessment of PI‐Al_2_O_3_ hybrid encapsulation strategy by accelerated aging of interdigitated electrodes (IDEs). a) 3D illustration of the layer structure of the IDEs. b) Optical microscope image of the IDE pattern. c) SEM cross‐sectional view of the PI/Al_2_O_3_/Ti/Au/Al_2_O_3_/PI structure in a flexible device after 120 days of aging at 57 °C. d) Photograph of a fabricated flexible IDE device already delaminated from the Si wafer. e) Impedance magnitude at 0.1 Hz and 1 kHz for PI‐Al_2_O_3_‐PI IDEs measured in different conditions (in PBS, in PBS with 3% H_2_O_2_, and in PBS with an applied 4 V DC voltage). Symbols represent the mean value of *n* = 4 sets of IDEs for each measure condition, along with the standard error markers. f) Schematic of the accelerated aging setup and connection of the IDE device to the potentiostat. g) Mean impedance magnitude (± standard error) vs frequency in PBS for 4 sets of IDEs for the same devices shown in (e), measured at specific intervals during accelerated aging. h) Average leakage current (dots) and standard error (shade) for the same devices as in (e) and (g).

**Figure 2:**
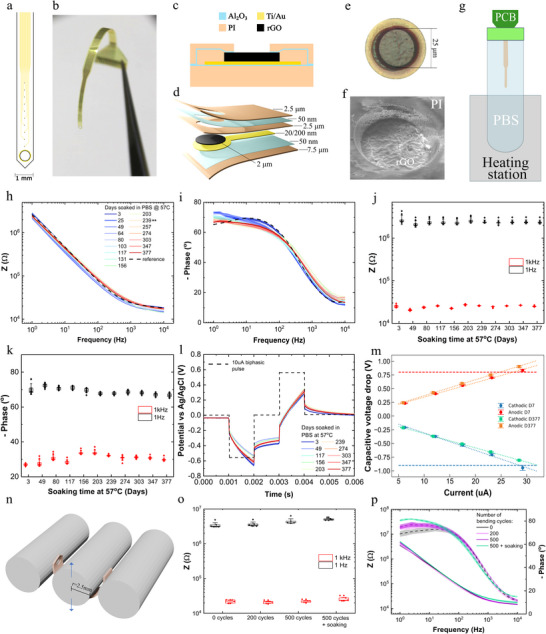
Accelerated aging of rGO microelectrodes incorporating PI‐Al_2_O_3_ encapsulation layers. a) Schematic of the linear rGO microelectrode array used to test long‐term stability, featuring 10 microelectrodes of 25 µm in diameter. b) Photograph of the flexible neural interface. c) Cross‐sectional schematic of an individual electrode, color‐coded by its structural layers. d) 3D illustration of a section of the device shown in panels (a) and (b), highlighting the sequential arrangement of the constituent layers; the numbers indicate the thickness of each layer. e) Optical microscopy image of a single electrode. f) SEM image of a single electrode. g) Accelerated aging setup used for all devices in this study. h) Mean impedance magnitude (± standard error, shaded area) vs frequency, measured over time in PBS at 57 °C, for *n* = 10 microelectrodes. The double asterisk (**) indicates the time point at which the number of functional electrodes were reduced from *n* = 10 to *n* = 9. i) Mean phase (± standard error) vs frequency over time, color‐matched with panel (g). j) Impedance magnitude at 1 Hz (black) and 1 kHz (red) vs soaking time, corresponding to the data in panel g. The boxplots indicate the 25th and 75th percentiles, with the median represented by a central line and markers denoting the standard error. Individual electrode data points are shown as dots. k) Phase magnitude at 1 Hz (black) and 1 kHz (red) vs soaking, corresponding to the data in panel (h). l) Mean voltage polarization (± standard error) vs time in PBS over the soaking time in days at 57 °C for the same microelectrodes as in panels (h–k). The dashed line represents the applied current pulse with an amplitude of 10 µA and a pulse width of 1 ms. m) Capacitive voltage drop of the voltage response during application of current pulses vs the applied current amplitude of pulses. The circles and squares represent the cathodic (blue, green) and anodic (red, orange) capacitive voltage drops at days 7 and 377, respectively. The error bars represent the standard deviation. The dashed blue and red lines represent the water window limits of the nanoporous graphene material of the electrodes, −0.8 V (blue) and 0.9 (red). The dotted lines (blue, green, orange, and red) represent the linear regression model fitted to the data to calculate the charge injection limit. n) Bending setup used to perform multi‐cycle mechanical bending of the flexible electrodes. o) Impedance magnitude at 1 Hz (black) and 1 kHz (red) after 200 and 500 bending cycles, followed by 10 days of soaking in PBS at 57 °C for *n* = 5 electrodes. p) Mean impedance (± standard error) (solid line) and mean phase (± standard error) (dashed line) magnitude vs frequency after 200 and 500 bending cycles followed by 10 days of soaking in PBS at 57 °C for *n* = 5 electrodes.

Two types of flexible devices were designed and fabricated: IDEs and flexible microelectrode arrays based on nanoporous rGO.^[^ ^3^, ^8^, ^28^, ^29^ ^]^ The IDEs (**Figure** **1**) were specifically used to assess the hermeticity of the encapsulation during accelerated aging experiments. Each IDE device has 10 pads for connection to a zero insertion force connector. The first and last connecting pads of the devices are connected via a continuous metal track to allow evaluation of the electrical connection throughout the experiments. The middle pads connect 4 sets of IDEs; each IDE is designed with 50 µm separation between fingers and 20 µm finger width (Figure 1b). IDEs with two different designs were prepared, for their different sensitivity to detect water ingress: one design, with a total device area of 7.3125 mm^2^, corresponding to a length of 9.75 mm and a width of 0.75 mm; another design, with an area of 3.6375 mm^2^, corresponding to a length of 4.85 mm and a width of 0.75 mm. Dimensions were chosen in order to obtain measurable impedance with standard potentiostats. Additionally, functional flexible graphene‐based microelectrode arrays were designed and fabricated (**Figure** **2**). The flexible microelectrode array consists of 10 microelectrodes of 25 µm diameter in a linear arrangement (Figure 2a), with a distance between electrodes of 450 µm. The 10 microelectrodes were located at the tip of the flexible device. The tip´s width is ≈1 mm and the total length of the device is 4 cm.

All devices were fabricated on a 7.5 µm thick polyimide (PI‐2611, HD MicroSystems) layer, spin‐coated onto a Si/SiO_2_ (500 µm, 285 nm) wafer, and baked at 350 °C in a nitrogen‐rich atmosphere. A 50 nm thick Al_2_O_3_ layer was deposited at 150 °C using atomic layer deposition (ALD) (Cambridge NanoTech Savannah S200 ALD), with the following parameters: Trymethylaluminum (TMA) pulse width = 0.01 s, H_2_O pulse width = 0.045 s, N_2_ flow = 20 sccm, base pressure = 0.7 Torr. After each precursor pulse, an idle step of 5 s was added. Next, metal layers were deposited: 20 nm of titanium (Ti) was deposited as an adhesion layer, followed by 200 nm of gold (Au), both deposited using an electron beam evaporation system (AJA Orion). For the microelectrodes, a nanoporous graphene‐based membrane of ≈2 µm thickness was prepared as described previously,^[^ ^3^ ^]^ and transferred onto the Au layer via a wet transfer technique followed by hydrothermal reduction. Aluminium (Al) was evaporated to define the membrane area at the electrode sites, and it was patterned using optical lithography (nLOF 2070, Microchemicals) and lift‐off (NI555). The graphene membrane was patterned using oxygen in an inductively coupled plasma reactive ion etcher (ICP‐RIE, Oxford PlasmaPro 100 Cobra), with the Al layer acting as a hard mask. Optical lithography using a positive photoresist (HIPR 6512, FujiFilm) and chemical wet etching processes were employed to pattern the Ti/Au bilayer. After metal patterning, IDEs were coated with an additional 50 nm Al_2_O_3_ layer; in the microelectrode array design, on the other hand, Al_2_O_3_ coating was done after spin‐coating a 2.5 µm thick PI‐2611 layer. The thickness of 2.5 µm was chosen to properly cover the patterned rGO membrane, which has a thickness of ≈2 µm. This PI layer was subsequently patterned using ICP‐RIE to expose electrode sites and portions of the device's outline and pads. To complete device encapsulation, all devices were spin‐coated with a final 2.5 µm‐thick PI‐2611 layer. A series of photolithography (using a thick positive photoresist‐AZ10XT, Microchemicals) and etching steps using ICP‐RIE were employed to pattern the PI‐Al_2_O_3_ layer structure, exposing electrode sites and connection pads, as well as etching the device outline for eventual delamination from the wafer. Specifically, the PI was etched using ICP‐RIE employing oxygen and argon as reactive gases. Finally, the aluminium on the electrodes was chemically etched using an aqueous etching solution of orthophosphoric acid, nitric acid, and deionized water (74% v/v H_3_PO_4_, 2.5% v/v HNO_3,_ 2.5% H_2_O). After fabrication, devices were manually separated from the supporting silicon wafer and inserted into custom printed circuit boards using ZIF connectors. The schematic of the detailed fabrication process can be found in Figure S5 (Supporting Information).

### 2.2 Experimental Setup

The samples (IDEs and rGO microelectrode arrays) were sealed with thermofusible glue inside plastic beakers containing a PBS solution (see next section), and were placed in a digitally controlled heating station (IKA, Dry block heater 2), as shown in Figure 2g. The devices were characterized and monitored periodically by means of electrochemical impedance spectroscopy (EIS), cyclic voltammetry (CV), chronoamperometry (current pulsing), and leakage current monitoring (LCM). The EIS and CV measurements as well as the characterization with current pulses for the microelectrodes were performed with an Autolab (Metrohm) potentiostat using a standard Ag/AgCl reference electrode and platinum (Pt) counter electrode, while the EIS measurements of the IDEs were performed with a Biologic potentiostat, due to its low current configuration; for this particular test configuration, the reference and counter electrode connectors of the potentiostat were shorted to one of the IDEs fingers, and the working electrode connector to the other IDE finger (Figure 1f). LCM of the IDEs was performed utilizing a Keithley 2400. Continuous DC biasing with 4 V was performed for a set of IDEs utilizing a Kethley 2450, where 4 terminals (1 of each set of IDEs) were short‐circuited and connected to one terminal of the Keithley while the remaining 4 terminals were connected to the other terminal. All the measurements were averaged for the 4 IDEs sets of each device.

The microelectrodes were subjected to stimulation with current pulses using a Neuronexus X‐Series XDAQ Core Family neurostimulator and an XDAQ Smartbox Pro X3SR headstage, which carries an Intan RHS 2000 chip. The applied biphasic current pulses had 100 width, 100 µs of interphase delay; we used a current amplitude of 50 µA, which corresponds to a charge injection level of 1 mC cm^−2^. Between the biphasic current pulses, discharging was enabled (using an option of the XDAQ system) to avoid continuous charging and, thus, faradaic reactions at the electrode/electrolyte interface. A stimulation frequency of 1 kHz was used (in contrast to the standard 130 Hz of DBS) to allow accelerated experiments. The stimulation was interrupted at selected time intervals only to perform EIS measurements. CV was performed only at the end of the stimulation for the electrodes stimulated with 1 mC cm^−2^.

Raman spectroscopy and scanning electron microscopy (SEM) were used to further characterize the devices under investigation. Raman spectra were acquired using a Witec spectrograph (Al‐pha300R) equipped with a 488 nm excitation laser and a 600 grooves/nm grating. Specifically, the Raman spectra were obtained by scanning a 10 × 10 µm^2^ area within the electrode to generate a spatial map. The SEM images were acquired using a ZEISS AIGURA 40, able to perform Focused Ion Beam (FIB) milling and equipped with an EDX detector.

### 2.3 Accelerated Aging Protocol

Accelerated aging conditions were used to expedite the determination of the lifetime of the proposed hybrid encapsulation by elevating the temperature, which is expected to accelerate degradation mechanisms.^[^ ^36^ ^]^ For degradation‐related reactions following first‐order kinetics, this increase is predictable, and the “aging factor” can be estimated. In our study, the temperature used to age the devices was 57 °C. To estimate the expected lifetime of the devices at 37 °C, the accelerated aging factor can be calculated using Arrhenius´ law, as previously described in other studies.^[^ ^37^ ^]^ This empirical relation has been found relevant for predicting degradation of polymers for biomedical applications, at least in the range up to 60 °C; higher temperatures might initiate processes that are not present in physiological temperatures.^[^ ^36^, ^38^ ^]^ For instance, Rubehn et al.^[^ ^34^ ^]^ showed that some polyimides (including PI‐2611), experienced loss of mass and a decrease in their mechanical properties when stored at temperatures higher than 60 °C. Given the selected conditions of our experiments and following previous reports,^[^ ^20^, ^33^ ^]^ the estimated aging factor is equal to 4. The aqueous environment we used is 1x PBS (phosphate‐buffered saline tablets, Sigma‐Aldrich). In some experiments involving devices with interdigitated electrodes, we added 3% H_2_O_2_ to simulate an oxidative environment in the brain. However, this was not included in all experiments, since there are indications that the addition of H_2_O_2_ can create a far harsher environment compared to a real biological environment.^[^ ^39^ ^]^ The PBS in all test beakers was emptied and refilled every two weeks.^[^ ^37^, ^39^, ^40^ ^]^

### 2.4 Bending Setup

The bending tests were performed using a 3D‐printed bending system carrying a 5 mm diameter rod that moves perpendicular the device's surface (Figure 2n) while the rest of the device is supported by two adjacent rods (also 5 mm diameter) placed close to the moving rod. The strain e (%) was calculated using e = h/2R,^[^ ^41^ ^]^ where h is the thickness of the tested film and R is the bending radius, which in this case is the radius of the bending rod.

## 3 Results

### 3.1 Stability Evaluation of the PI‐2611/ Al_2_O_3_ Hybrid Encapsulation

The stability of the hybrid PI/Al_2_O_3_ encapsulation was first evaluated using fully flexible devices with interdigitated electrode patterns (see Figure 1). IDEs (20 µm finger width and 50 µm separation between fingers, see Figure 1b) were fabricated as described in the Methods section. Elevated temperature accelerated aging conditions were used to expedite the determination of the lifetime of the hybrid encapsulation. To this end, devices were immersed in PBS, or PBS with 3% H_2_O_2_, at a temperature of 57 °C. Additionally, to assess the encapsulation's performance under continuous electrical stress, a group of IDEs was continuously biased with 4 V DC voltage.

EIS and leakage current measurements were periodically performed, as described in the Methods section. Figure 1e,g,h summarizes the impedance results of the tested samples. All samples exhibit capacitive behavior in the investigated frequency range, as evidenced by the impedance spectra (Figure 1g). For completeness, the phase of the impedance data is provided in the supplementary material (Figure S1, Supporting Information). The stability of the impedance measurements over the course of the aging test is demonstrated in Figure 1e, which depicts the impedance magnitude at 0.1 Hz and 1 kHz for all tested samples. While the 1 kHz impedance is a typical figure of merit used to benchmark the performance of neural interface electrodes, we have included the impedance at low frequency (0.1 Hz) since it can be used to assess the presence of resistive paths in case of encapsulation failure. Figure 1e shows that both the 0.1 Hz and 1 kHz impedance remained stable over time; the minor fluctuations observed for the impedance at 0.1 Hz are attributed to the noise inherent to the measurement of such high impedance values (10^11^ Ω).

Our impedance data show that unbiased samples remained stable for the whole duration of the study: 609 days for the IDEs soaked in PBS and 579 days for the IDEs soaked in PBS with 3% H_2_O_2_. Biased samples at 4 V were assessed for a shorter period; yet, they remained stable for over 320 days, the maximum time they were tested. The leakage current measurements (Figure 1h) further support the durability of the devices. Despite some minor noise‐related perturbations, leakage current remained well below the 1 nA threshold throughout the aging test. A current exceeding this threshold would be considered indicative of encapsulation degradation. Complementing the electrochemical characterization, we performed structural characterization, using SEM on FIB‐cross‐sections (Figure 1c), to prove the stability of the encapsulation layer for aged devices (in this case for 120 days in PBS at 57 °C). In addition, a SEM cross‐sectional view of IDEs soaked for 552 days can be found in Figure S4 (Supporting Information).

Overall, the electrical and structural evaluation conducted confirms the encapsulation integrity for all tested devices, validating that the PI‐Al_2_O_3_ strategy can provide robust long‐term protection for water ingress.

### 3.2 Stability Assessment of Neural Arrays of Nanoporous Reduced Graphene Oxide Electrodes with PI‐Al_2_O_3_ Hybrid Encapsulation in Saline Solution

Microelectrode arrays with nanoporous rGO as electrode material were fabricated, integrating the PI/Al_2_O_3_ encapsulation strategy. The design of the neural microelectrode arrays used in this study is shown in Figure 2a–d. Details of the microfabrication are provided in the Methods section. The full fabrication process is depicted in Figure S5 (Supporting Information). The devices feature a linear array of 10 electrodes of 25 µm diameter, with an inter‐electrode distance of 450 µm. The tip of the device has a hole intended for guiding the deep implantation of this probe in the brain of a large animal model. As shown in the device cross‐section and exploded‐view (Figure 2c,d), the device consists of three polyimide layers and two layers of Al_2_O_3_. The first Al_2_O_3_ layer was deposited before the metal of the tracks, while the second was deposited immediately after spin coating the intermediate polyimide layer right on top of the electrode material. An intermediate polyimide layer was incorporated to align with the current advancements in encapsulation strategies for implantable bioelectronics: it has been shown that increasing the complexity of the diffusion path to the metallic tracks enhances barrier performance and improves device longevity.^[^ ^9^, ^21^ ^]^

For the long‐term assessment in vitro, the nanoporous rGO microelectrodes were left to soak in a sealed plastic beaker with PBS at 57 °C and were taken out of the beaker at regular time intervals to be characterized electrochemically. The setup of the electrochemical characterization is shown in Figure 2g. Figure 2h–k compile the impedance measurements over 377 days, revealing that the impedance magnitude remains in the range 20–30 kΩ at 1 kHz and 2–3 MΩ at 1 Hz.

In addition to the impedance characterization, we also analyzed the response of the microelectrodes to biphasic current pulses during the long‐term aging study. Figure 2l depicts the voltage response of an electrode to a biphasic current pulse of 10 µA amplitude and 1 ms pulse width (corresponding to a charge injection of 2 mC cm^−2^); as the figure shows, the voltage response remained consistent in shape and amplitude along the testing period. In order to determine the charge injection limit (CIL) of the electrodes, which quantifies the maximum charge that can be delivered without exceeding water electrolysis limits, we applied biphasic current pulses with increasing amplitude (from 5 to 30 µA) and a pulse width of 1 ms. To determine the CIL, we first calculated the capacitive voltage drop observed for each pulse amplitude for both the anodic and the cathodic phase, which corresponds to the response that follows the initial sharp step‐like response (ohmic drop) and appears as an increasing voltage during the pulse (see Figure 2l). The capacitive voltage drop results from the charging of the electrode–electrolyte capacitive interface in response to the injection of a constant current. Anodic and cathodic CIL were calculated as the intersection of the linear regression of the capacitive voltage drops at different applied currents and the voltage limits after which faradaic reactions occur at the nanoporous rGO electrode‐electrolyte interface, which correspond to the horizontal dotted lines in Figure 2m. The limits are 0.8 V vs Ag/AgCl for the anodic phase and −0.9 V vs Ag/AgCl for the cathodic phase, and were established from previous works employing the same electrode material.^[^ ^3^, ^28^ ^]^ In addition to electrochemical performance monitoring, optical images of the microelectrodes were acquired after 151 days of soaking (Figure S7, Supporting Information), further confirming their structural integrity and long‐term stability.

Interestingly, there is an apparent slight improvement in the CIL in the cathodic domain and a slight decrease in the CIL in the anodic domain. Specifically, in the cathodic domain, the CIL increased from 5.8 mC cm^−2^ on day 7 to 6.5 mC cm^−2^ on day 377, corresponding to a 12.1% change. On the other hand, the CIL in the anodic domain decreased from 5.5 mC cm^−2^ on day 7 to 5 mC cm^−2^ on day 377, corresponding to a −10% change. Overall, the electrodes show good stability for 377 days at 57 °C (equivalent to 1508 days at 37 °C, assuming an acceleration factor of 4) and maintain a high charge injection capacity (over 5 mC cm^−2^).

The mechanism behind the observed modest change in CIL following long‐term aging, opposite shifts in anodic and cathodic CIL, is not fully understood. We tentatively relate it to changes in surface chemistry or ion transport within the pores of the rGO‐based material. Soaking in a wet environment can alter oxygen‐containing functional groups and pore accessibility when considering the porous nature of this material, which in turn modulates the pseudocapacitive performance of the electrode.^[^ ^28^ ^]^

To complement the robustness assessment of the fabricated neural interfaces, mechanical bending tests were performed using a customized 3D‐printed system, providing a bending radius of 2.5 mm. The tests were conducted by flexing the device's electrode‐carrying part by moving the rod perpendicular to the device's surface (Figure 2n), with the electrodes facing downward. Considering a total encapsulation thickness of h = 12.5 µm, the strain was calculated (see Section 2.4) to be 0.25%. The bending test was followed by a soaking period of 10 days (PBS, 57 °C). The accelerated aging step was added to expose potential failures in the hybrid PI/Al_2_O_3_ encapsulation as a result of the bending steps, such as the formation of microcracks or delamination.

The impedance spectra recorded after 200 and 500 bending cycles, as well as following a 10‐day soaking period, showed no evidence of performance deterioration. No delamination of the rGO active layer was observed, which would otherwise shift the spectra to higher impedance values. Similarly, no reduction in low‐frequency impedance, typically associated microcrack‐induced leakage pathways between the electrode and the electrolyte or across adjacent electrodes, was detected.

### 3.3 Stability Assessment of the Neural Arrays Under Continuous Long‐Term Electrical Stimulation

**Figure 3:**
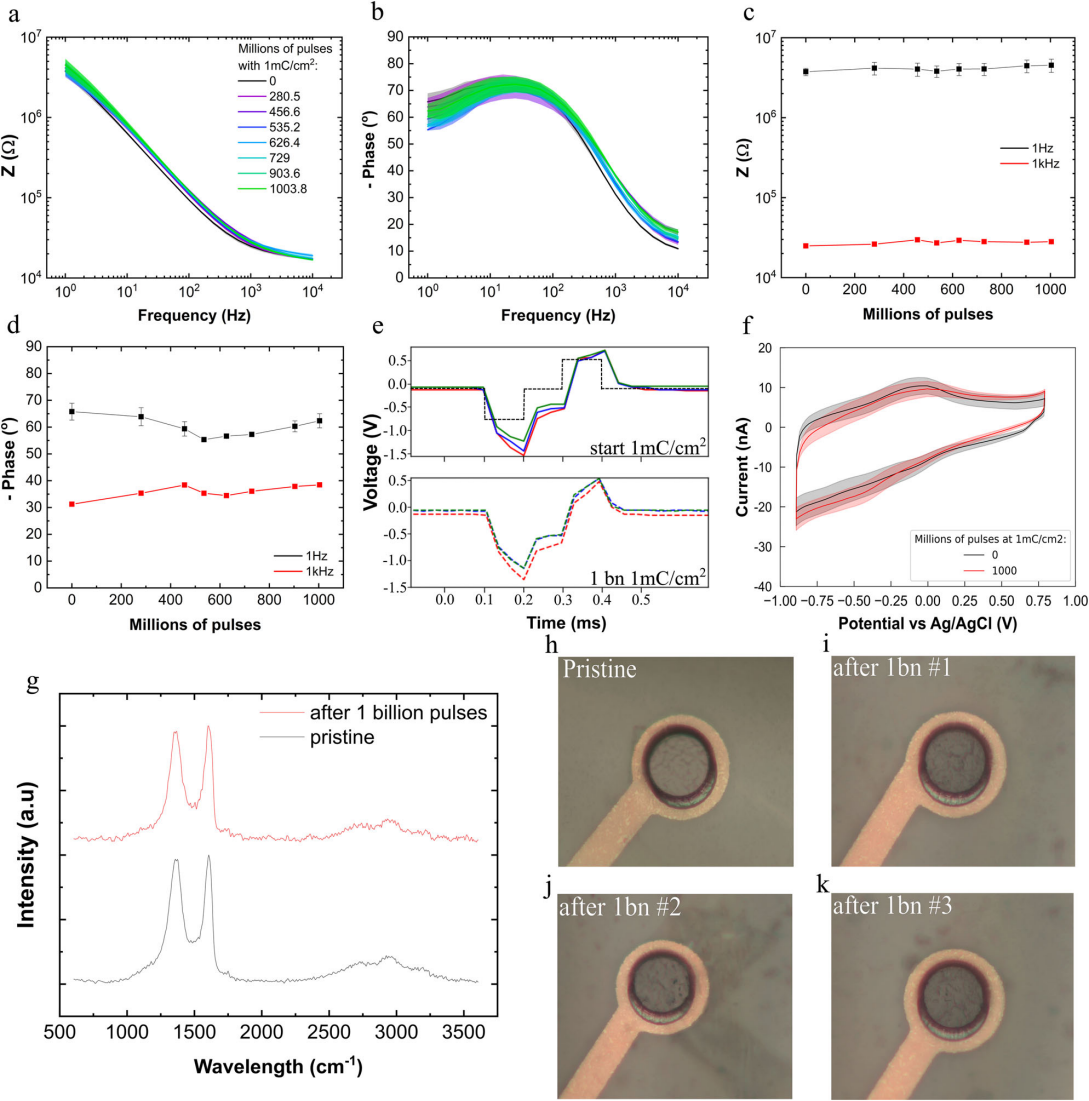
Assessment of rGO microelectrodes during accelerated stimulation. a,b) Mean impedance magnitude (panel a) and phase (panel b) vs frequency of rGO microelectrodes in PBS during the long‐term simulation study with a charge injection of 1 mC/cm^2^ (biphasic current pulses of 100 µs pulse width, 100 µs interphase delay, 50 µA pulse amplitude, 1 kHz stimulation frequency). c,d) Mean (± standard error) impedance magnitude (c) and phase (d) at 1 Hz (black) and 1 kHz (red) vs the number of pulses (in millions). The data correspond to *n* = 3 electrodes. e) Electrode voltage polarization in response to the current stimulation pulses (solid lines: before stimulation study; dashed lines: after stimulation study) for *n* = 3 electrodes (red, yellow, and blue). The black dashed line in the top plots represents the applied current pulse. f) Cyclic voltammograms recorded in PBS before and after 1 billion pulses at 1 mC cm^−^^2^. g) Raman spectra of a pristine electrode (black) and an electrode after stimulation with 1 billion pulses (red). h–k) Optical microscopy images of a pristine electrode (panel h) and three different electrodes (panels i‐k) after 1 billion pulses at 1 mC cm^−^^2^.

To further validate the stability of the fabricated nanoporous graphene electrodes with the hybrid PI‐Al_2_O_3_ encapsulation, we subjected them to continuous stimulation using biphasic current pulses of 100 µs pulse width, 100 µs interphase delay, amplitudes of 20 and 50 µA, and 1 kHz stimulation frequency. While typical stimulation frequencies for DBS are close to 100 Hz, we have chosen 1 kHz to accelerate the stimulation study. Between the biphasic pulses, a discharging step was included to avoid charge accumulation and thus unwanted polarization (see Methods section). The two pulse amplitudes used in our study correspond to charge injections of 0.4 and 1 mC cm^−2^ per phase. This charge density is well above the charge density limit approved for clinical devices, which is 30 µC cm^−2^ per phase.^[^ ^11^, ^42^ ^]^ **Figure** **3** summarizes the electrochemical, optical, and structural characterization of the electrodes after 1 billion pulses at 1 mC cm^−2^ (data for the 0.4 mC cm^−2^ can be found in Figure S8, Supporting Information). In Figure 3a–d, no significant differences are observed in the impedance or phase spectra before, during and after 1 billion stimulation pulses. Minor variations observed in the phase response are attributed to slight experimental perturbations or inconsistencies between successive measurements. We also assessed the electrodes´ polarization during the long‐term stimulation study. Figure 3e depicts exemplary voltage responses at the electrodes upon the stimulation pulses, evidencing that the voltage responses were similar before and after the stimulation experiments. Additionally, cyclic voltammetry measurements of the electrodes (Figure 3f) conducted before and after 1 billion pulses at a charge injection level of 1 mC cm^−^^2^ demonstrate minimal differences.

In addition to the electrochemical characterization following the stimulation experiments, the electrodes were visually inspected and examined using Raman spectroscopy. Figure 3g depicts exemplary Raman spectra of microelectrodes obtained before and after stimulation, corresponding to the average of 10 × 10 µm^2^ area within the electrode. Both the I_D_/I_G_ ratio (related to the density of defects in graphene)^[^ ^43^ ^]^ and the I_D+G_/I_D_ ratio (related to the reduction level of rGO)^[^ ^44^ ^]^ remain constant before and after stimulation, indicating no significant difference in terms of the reduction level of the rGO or in terms of defect generation. Furthermore, optical inspection of a pristine electrode and three electrodes stimulated at 1 mC cm^−^^2^ (Figure 3h–k) reveals no visible signs of degradation in the stimulated electrodes. Thus, our data indicate that the continuous stimulation (1 billion pulses at 1 mC cm^−2^) of the rGO microelectrodes does not induce any relevant change in the electrode performance or in the material structure and chemical composition.

## 4 Discussion and Conclusion

Bidirectional thin‐film neural interfaces demand advanced encapsulation strategies that are simultaneously thin, mechanically robust, and stable over long durations. At the same time, the use of novel electrode materials with low impedance, high charge injection capacity, and excellent biocompatibility is essential to ensure sustained functionality over extended periods.

Here, we present the integration of nanoporous rGO microelectrodes with a hybrid organic‐inorganic encapsulation strategy based on polyimide and ALD‐deposited Al_2_O_3_, demonstrating long‐term stability of this flexible neurotechnology under accelerated conditions. The water barrier performance of the PI‐Al_2_O_3_ encapsulation was evaluated via electrochemical impedance spectroscopy and leakage current monitoring using interdigitated electrodes immersed in phosphate‐buffered saline at 57 °C. The encapsulation remained stable throughout the entire testing period, 609 days at 57 °C (equivalent to ≈6.6 years at 37 °C) under unbiased conditions, and 321 days (equivalent to 3.5 years at 37 °C) under continuous 4 V DC bias. To assess the long‐term functionality of the nanoporous rGO microelectrode technology integrating the PI‐Al_2_O_3_ encapsulation, we periodically monitored electrochemical performance while flexible arrays with microelectrodes were soaked in phosphate‐buffered saline at 57 °C. The encapsulated rGO microelectrodes retained stable electrochemical characteristics with no observable degradation for 377 days at 57 °C (approximately equivalent to 4.1 years at 37 °C). Furthermore, the mechanical robustness of the encapsulated electrodes was assessed by performing bending tests using 0.25% strain. The electrochemical performance of the electrodes remained stable after 500 bending cycles plus 10 days of soaking in PBS at 57 °C. Finally, the same microelectrodes withstood 1 billion biphasic current pulses (1 mC cm^−2^) without noticeable changes in electrochemical performance (impedance or charge injection limit), underscoring their durability for chronic neural interfacing applications.

Numerous studies have investigated inorganic layers to enhance the water barrier characteristics of thin‐film neurotechnology (Table S1, Supporting Information). Testing methodologies vary from interdigitated electrodes (IDEs)^[^ ^22^, ^24^ ^]^ and metal plates^[^ ^20^ ^]^ to magnesium tracks,^[^ ^21^ ^]^ often using different accelerated aging conditions, with lifetimes extrapolated for either 37 or 25 °C. Many of the studies, however, use rigid substrates (e.g., Si/SiO_2_,^[^ ^24^ ^]^ quartz^[^ ^22^ ^]^ or glass^[^ ^20^ ^]^), which do not fully represent flexible implants because they do not account for delamination pathways critical in flexible neural interfaces.^[^ ^10^, ^33^ ^]^ Li et al.^[^ ^20^ ^]^ achieved the longest reported lifetime using a PI‐HfO_2_/Al_2_O_3_/HfO_2_‐PI trilayer on a rigid‐support. Mariello et al.^[^ ^21^ ^]^ introduced Mg‐resistivity testing for a PI/(Al_2_O_3_/TiO_2_/ParC) dyad stack, reporting extrapolated lifetimes for devices of similar complexity as in this manuscript of up to 68 days (4 layers) and 1.3 years (5 layers) at 25 °C. Devices with a higher number of dyads exhibit stability of up to 6.4 years under the same conditions. A comparison between the aforementioned studies and this work can be found in Table S1 (Supporting Information). To further enhance hermeticity and reach several decades of stability or even full human life durability, strategies such as multiple stacks of organic‐inorganic layers might be required.^[^ ^21^ ^]^

Beyond assessing the stability of the encapsulation strategy in test structures, implementing and validating it in functional neural probes introduces new challenges, including multilayer fabrication, electrode openings, and mechanical flexing, each presenting different failure risks. Previous studies have demonstrated the stability of flexible neural interfaces over periods ranging from 6 weeks to a full lifetime of rodents in vivo and to more than two years in vitro, with stimulation protocols delivering millions to billions of pulses (see Table S2, Supporting Information).^[^ ^7^, ^11^, ^31^, ^32^, ^33^, ^45^, ^46^ ^]^

While several works reported stability beyond 1 billion pulses, very few combined this number of pulses with high levels of injected charge density. Oldroyd et al.^[^ ^33^ ^]^ achieved the longest in vitro stability, maintaining functionality for over 2 years in PBS at 37 °C using a PEDOT:PSS electrode encapsulated with polyimide and delivering more than 1 billion pulses. However, the injected charge per phase was limited to 0.032 mC cm^−^^2^, restricting its validation to low charge‐density stimulation. Schander et al.^[^ ^46^ ^]^ delivered 4.6 billion pulses using PEDOT:PSS electrodes over 386 days in PBS at 37 °C, with a charge injection of 0.203 mC cm^−^^2^. At even higher charge‐density, Böhler et al.^[^ ^7^ ^]^ demonstrated stability for over 1 billion pulses with IrOx electrodes at 1 mC cm^−^^2^, validated for 5 months in vivo. Lycke et al.^[^ ^32^ ^]^ also showed 8 months of chronic use of flexible IrOx electrodes for intracortical microstimulation, delivering 1.9 million pulses while maintaining reliable neural recording. Using carbon‐based electrodes, Vomero et al.^[^ ^45^ ^]^ achieved 1 million pulses with glassy carbon electrodes at 0.43 mC cm^−^^2^. By combining glassy carbon with PEDOT:PSS, they delivered 5 million pulses at a very high density of 10.6 mC cm^−^^2^ per phase. Schiavone et al.^[^ ^12^ ^]^ demonstrated stability for 1 billion pulses at 0.042 mC cm^−^^2^ using a Pt/PDMS composite and validated the technology in vivo for 6 weeks and in vitro for 110 days at 37 °C. Previous works featuring the same electrode material as in this study but with only PI encapsulation, achieved the application of 15 million and 100 million biphasic current pulses at 3 and 1 mC cm^−^^2^, respectively, while the maximum reported stability in vivo was 90 days.^[^ ^3^, ^8^ ^]^ In comparison, our flexible graphene‐based electrode arrays incorporating the PI‐Al_2_O_3_ encapsulation strategy showcase excellent stability for an extrapolated period of ≈1508 days at 37 °C. Under biphasic current pulses at 1 kHz and a charge density of 1 mC cm^−^^2^, microelectrodes endured 1 billion pulses without noticeable performance deterioration.

Our accelerated in vitro protocol does not account for biological factors such as protein adsorption, inflammatory responses, or tissue micromotion in vivo. Future work will involve chronic implantation studies to assess encapsulation integrity and recording/stimulation efficacy over time. Furthermore, regarding the characterization of the interface PI‐Al_2_O_3,_ future work will focus on extracting the crack‐onset strain for the PI‐Al_2_O_3_ hybrid encapsulation using a standardized fragmentation protocol and performing adhesion peeling tests to evaluate the adhesion strength for the PI‐Al_2_O_3_ interface, assessing in this way our encapsulation method for high‐strain applications. Furthermore, although a dedicated biocompatibility assessment has not yet been conducted, previous studies have demonstrated that flexible implants based on polyimide substrates with rGO microelectrodes exhibit excellent biocompatibility for brain and peripheral nerve applications.^[^ ^3^, ^47^ ^]^ While the devices presented in this work incorporate an Al_2_O_3_ layer, no adverse biocompatibility issues are anticipated, as only extremely small areas containing Al_2_O_3_ (at the very edge of the device) are exposed to biological tissue. Nonetheless, future investigations will specifically address the long‐term biocompatibility and biostability of these encapsulated interfaces.

Overall, our study establishes a scalable wafer‐scale, flexible thin‐film technology platform integrating hybrid PI‐Al_2_O_3_ encapsulation with nanoporous reduced graphene oxide microelectrodes, validating its long‐term durability under accelerated aging conditions, after mechanical bending, and under high charge density stimulation conditions. By integrating the rGO microelectrode technology with the PI‐Al_2_O_3_ encapsulation and demonstrating in vitro chronic stability, our work addresses key challenges in long‐term neuromodulation, advancing the use of graphene thin film technologies in next‐generation neural interfaces targeting lifelong medical implants.

## Conflict of Interest

J.A.G declares that he holds an interest in INBRAIN Neuroelectronics, which has licensed the technology described in this paper. All other authors declare no conflict of interest.

## Author Contributions

G.A.K. conducted all experiments, analyzed the data, and wrote the original draft; X.I. and N.R. contributed to the fabrication of the devices that were used in this study; E.d.C contributed to the Raman measurements; J.A.G. and E.M.C. led the design of the study and its supervision. All authors contributed to the manuscript preparation.

## Supporting information



Supporting Information

## Data Availability

All the data in the main text has been deposited at https://doi.org/10.34810/data2737. Supplementary experimental data that support the figures and other findings of this study can be obtained by contacting the corresponding authors. Authors can make data available on request, agreeing on the data formats needed.
