# Supplementary material for: Long‐Term Stable Neural Interfaces with Nanoporous Graphene Electrodes and Hybrid Polyimide‐Aluminium Oxide Encapsulation
Source: Small Methods. 2025 Nov 10;9(12):e01720. doi: 10.1002/smtd.202501720 (PMC12716225; doi:10.1002/smtd.202501720)
Supplement: Supplementary file 1 — Supporting Information [file SMTD-9-e01720-s001.docx]

Supporting Information

**Long-term stable neural interfaces with nanoporous graphene electrodes and hybrid polyimide-aluminum oxide encapsulation**

Georgios Alexandros Katirtsidis^1^, Xavier Illa^2,3^, Nicola Ria^1^, Elena del Corro^1,6^, Eduard Masvidal-Codina*^1,3,4,6^ Jose A. Garrido*^1,5^

1. Catalan Institute of Nanoscience and Nanotechnology (ICN2), CSIC and BIST, Campus UAB, Bellatera, Spain
2. Institute de Microelectronica de Barcelona IMB-CNM (CSIC), Campus UAB, Bellatera, Spain
3. Centro de Investigacion Biomedica en Red de Bioingeneria, Biomateriales y Nanomedicina, Instituto de Salud Carlos III, Madrid, Spain
4. Institute of Neurosciences, Universitat Autònoma de Barcelona, Cerdanyola del Vallès, Spain
5. ICREA, Barcelona, Spain
6. Spanish National Research Council (CSIC), Madrid, Spain

**Interdigitated electrodes electrical and structural characterization**

Figure S1 shows the impedance phase corresponding to the experiments shown in Figure 1 of the main manuscript. In the high frequency regime, the phase at 1kHz of all types of devices remains close to -90^o^, clearly indicating a capacitive behaviour during the whole period of the experiment. At 1 Hz, despite the noise perturbations, there is not a clear trend that would indicate degradation. For Figures 1 and S1, we attribute the noise perturbations in the lower frequency regime, to equipment limitations and the noise caused by 50 Hz coupling.


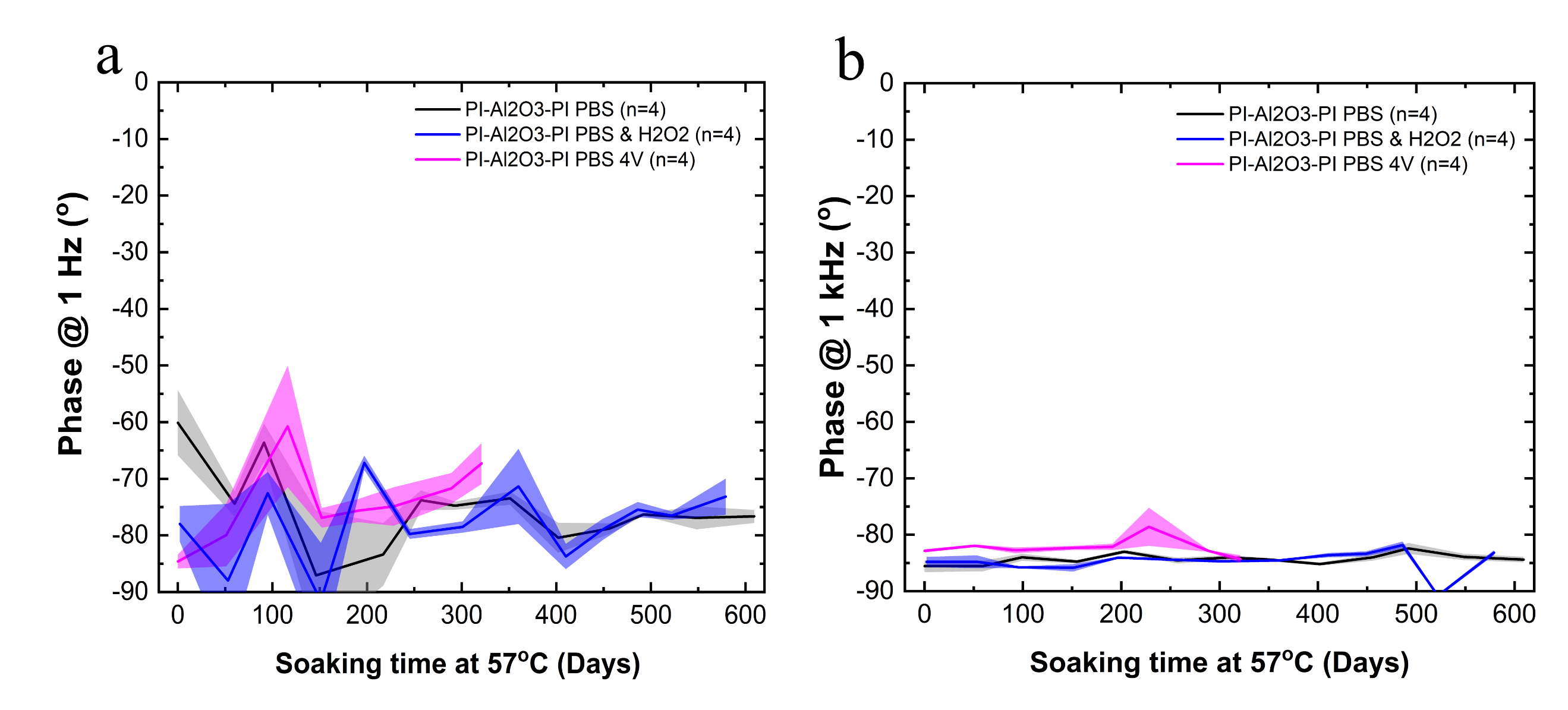


**Figure S1**. Mean phase (± standard error, shaded area) for 1 Hz (panel a) and 1 kHz (panel b) versus soaking time in PBS for 4 sets of IDEs for the same devices shown in Figure 1h, measured at specific intervals during accelerated aging.

For comparison and to further demonstrate the superiority of the proposed polyimide/Al₂O₃ hybrid encapsulation, we evaluated its performance against purely polymeric encapsulation using polyimide. As shown in Figure S2, two IDEs encapsulated only between polyimide layers without additional processing exhibited a rapid decrease in impedance magnitude at both 0.1 Hz and 1 kHz after just 2 days of soaking at 57 °C, primarily due to poor interlayer adhesion. Introducing a surface activation step (ICP-RIE treatment) followed by adhesion promoter spin-coating before the deposition of the second PI layer, significantly delayed degradation: impedance remained stable for approximately 100 days, after which a noticeable drop at low frequencies occurred, ultimately leading to catastrophic failure after approximately 150 days.

In contrast, IDEs encapsulated with the PI–Al₂O₃ hybrid barrier remained stable for at least 600 days under the same soaking conditions, while devices subjected to a 4 V DC bias also maintained stability for at least 300 days. These results clearly underscore the importance of combining polyimide with Al₂O₃ to achieve long-term stability, which is essential for chronically reliable implants and bioelectronic devices.


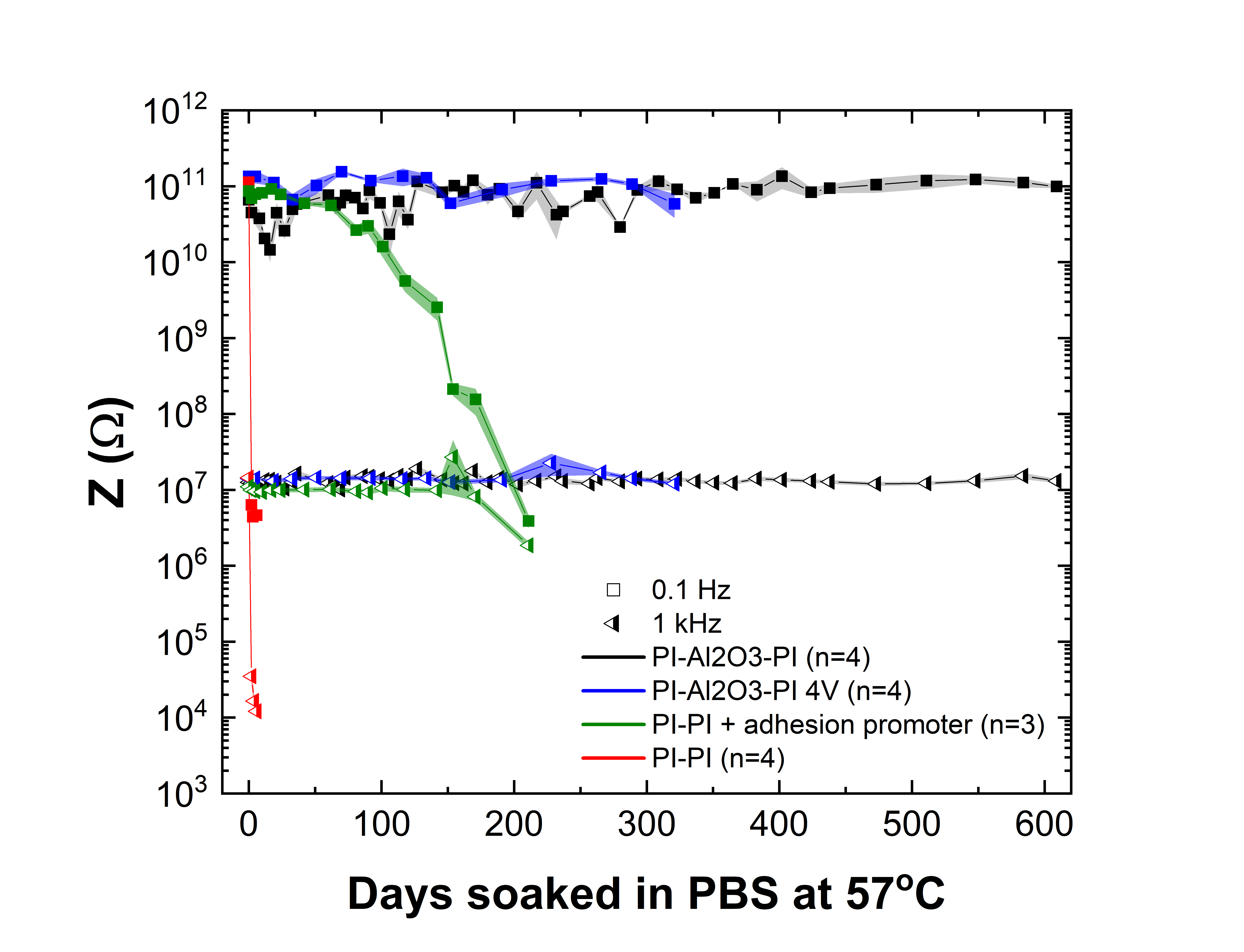


**Figure S2**. Mean impedance magnitude (± standard error, shaded area) for 0.1 Hz (squares) and 1 kHz (triangles) versus soaking times in PBS for IDEs with different encapsulations involving polyimide.

Complementary to the electrical characterization, we performed structural characterization of an IDE device aged for 120 days in PBS at 57^o^C using scanning electron microscopy (SEM). SEM images were acquired using ZEISS AIGURA 40, able to perform Focused Ion Beam (FIB) milling and equipped with an EDX detector.


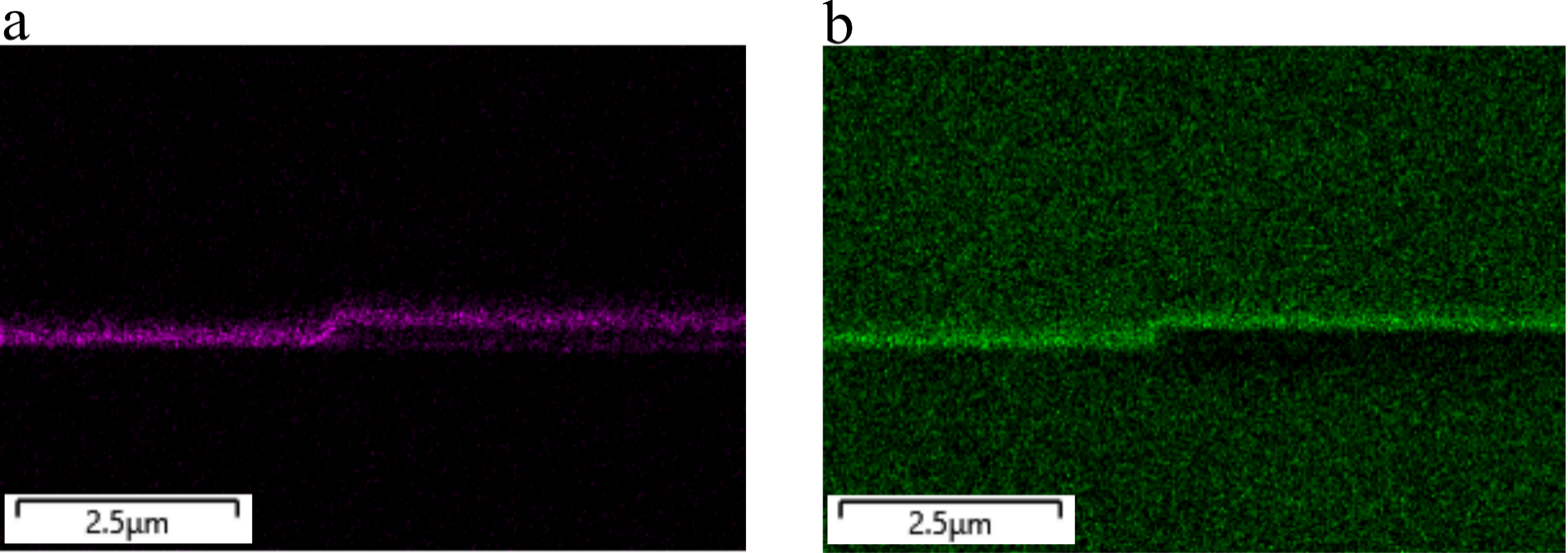


**Figure S3**. EDX analysis of the IDEs SEM cross-sectional view (Figure 1c). (a-b) Elemental maps of aluminium (a) and oxygen (b) acquired using Energy Dispersive X-ray Spectroscopy (EDX).


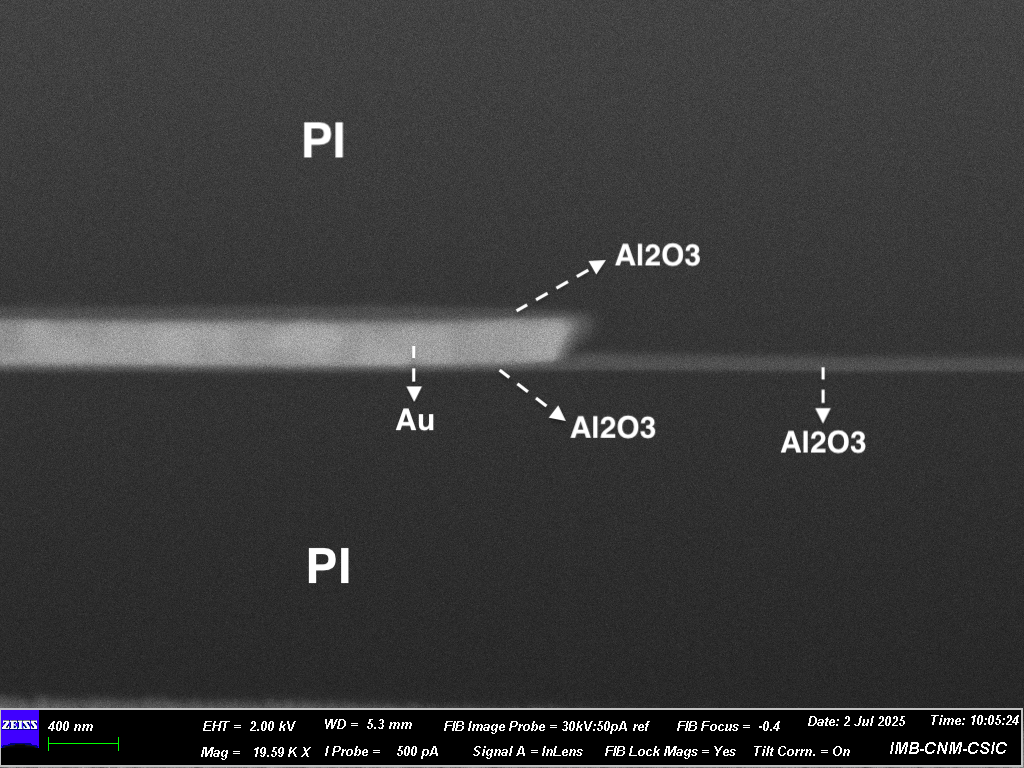


**Figure S4**. SEM cross-sectional view of IDEs soaked for 552 days in PBS at 57^o^C.

**Device fabrication**

The fabrication of the flexible microelectode arrays presented in this study is summarized in Figure S4. The fabrication of IDEs, was performed in a similar way, following only the steps shown in panels a-d, panels j-k which correspond to the patterning of the interdigitated metal traces, panels o-p, which correspond to the deposition of the second Al_2_O_3_/polyimide stack, panels q-r, which correspond to the etching of the top Al_2_O_3_/polyimide stack, panels s-t, which correspond to the cutting of the structure of the device and panel v for the delamination of the device from the substrate


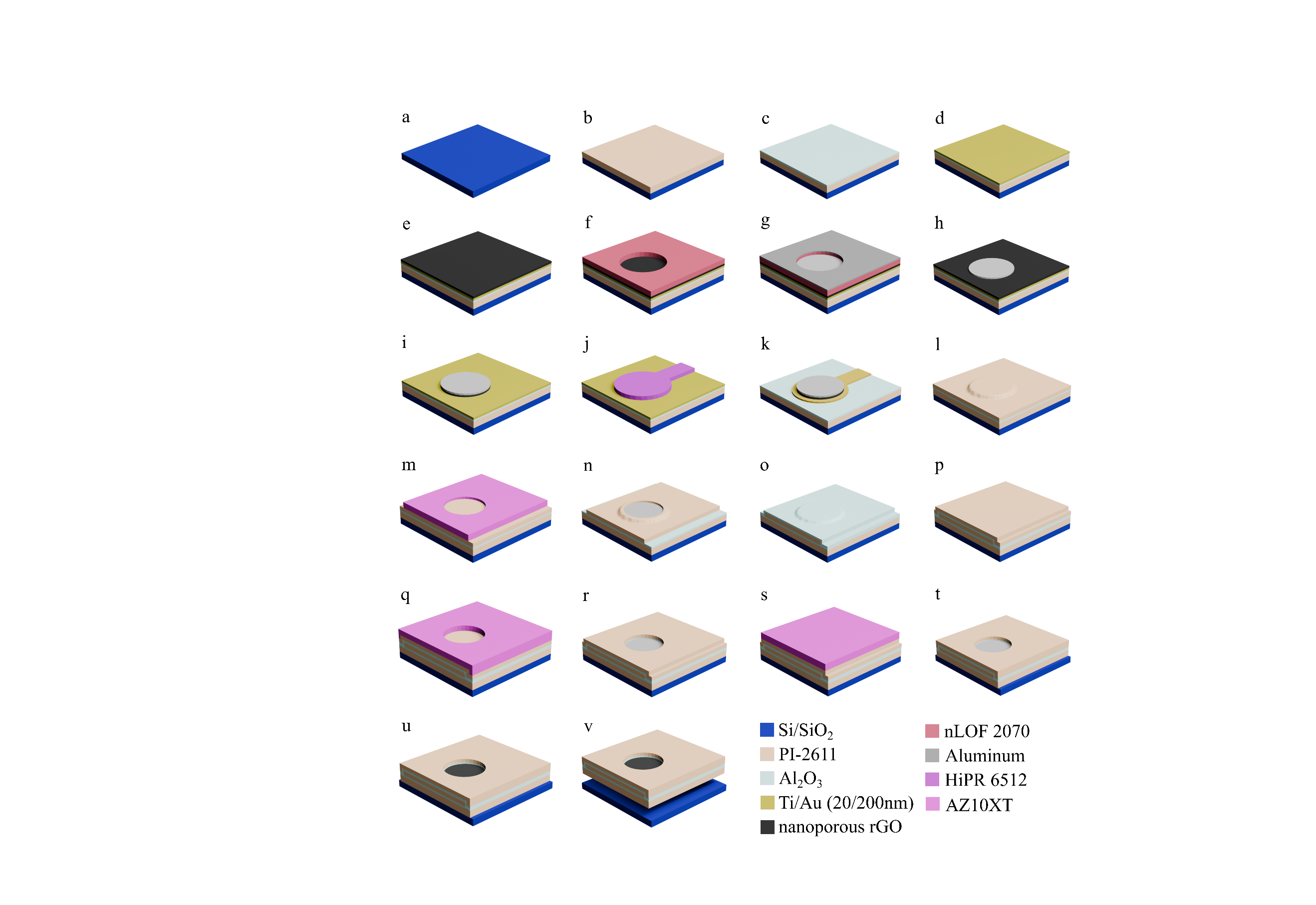


**Figure S5.** Fabrication process of the flexible rGO microelectrode arrays integrating the PI- Al₂O₃ encapsulation. (a) Devices were fabricated on 4″ Si/SiO₂ wafers (400 μm / 285 nm). (b) Spin-coating and hard-baking of a 7.5 μm polyimide layer (PI-2611, HD Microsystems). (c) Deposition of a 50 nm Al₂O₃ layer via atomic layer deposition (ALD). (d) Deposition of a 20/200 nm Ti/Au stack using e-beam evaporation. (e) Transfer of a 1 μm thick nanoporous rGO membrane. (f) Photolithography with nLOF 2070 negative resist to define the electrode area. (g) Evaporation of a 120 nm aluminium layer. (h) Lift-off of aluminium and resist removal. (i) Patterning of the rGO membrane using ICP-RIE. (j) Photolithography with HiPR 6512 positive resist to define metal traces. (k) Wet etching of Ti/Au and resist removal. (l) Spin-coating and hard-baking of the 2.5 μm thick intermediate polyimide layer. (m) Photolithography with AZ10XT resist to expose the electrode area. (n) Opening of the intermediate polyimide using ICP-RIE and resist removal. (o) Deposition of a second 50 nm Al₂O₃ layer via ALD. (p) Spin-coating and hard-baking of the 2.5 μm top polyimide layer. (q) Photolithography with AZ10XT resist to expose electrode sites. (r) Opening of the top polyimide and Al₂O₃ layers using ICP-RIE and resist removal. (s) Photolithography with AZ10XT to define the overall device shape. (t) Etching of the polyimide and Al₂O₃ down to the Si/SiO₂ substrate using ICP-RIE, followed by resist removal. (u) Wet etching of the aluminium layer (see Methods section). (v) Device release from the substrate via mechanical delamination.

**
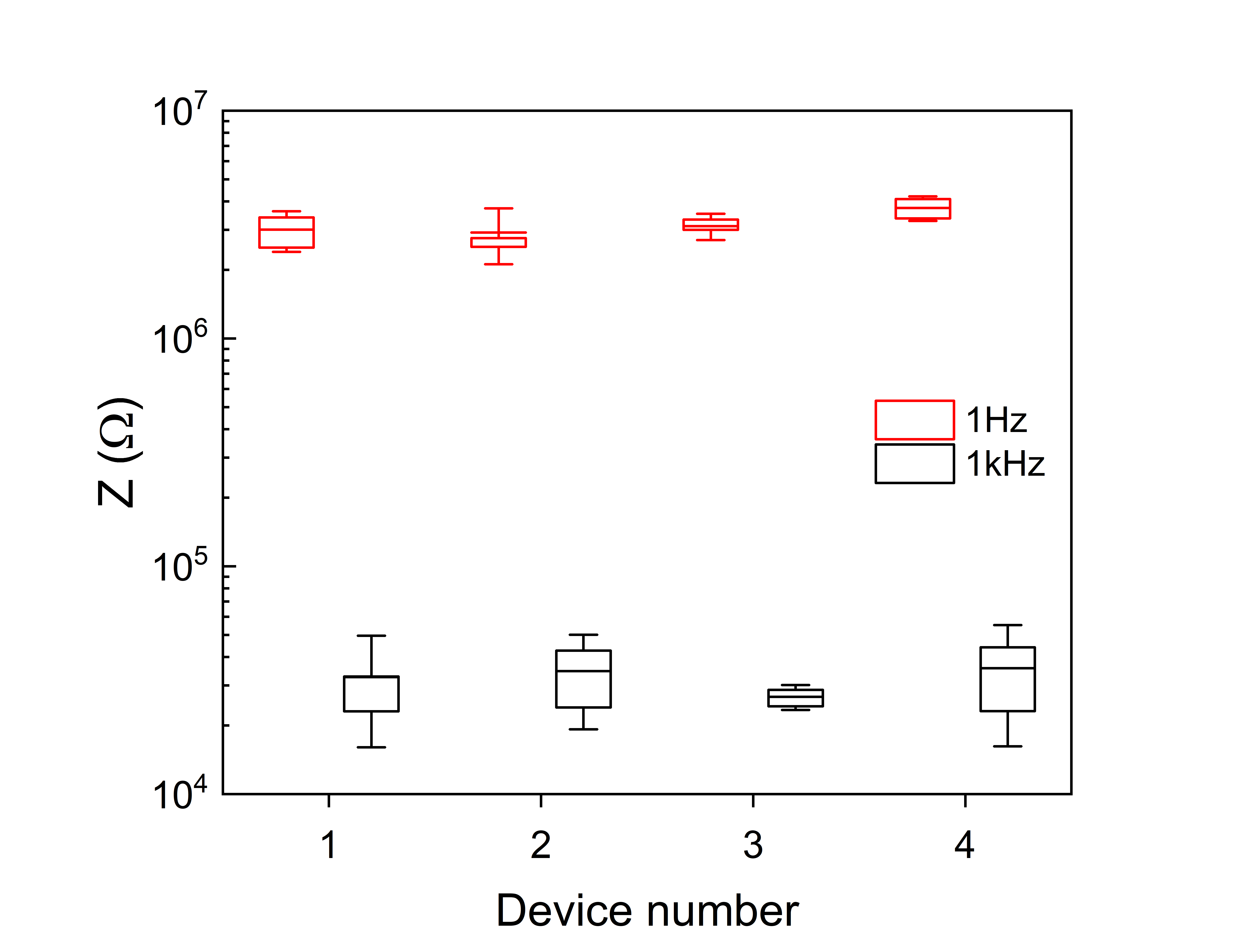
**

**Figure S6.** Impedance magnitude at 1 Hz (red) and 1 kHz (black) of four arrays. The boxplots indicate the 25th and 75th percentiles, with the mean represented by a central line and markers denoting the standard deviation.

**Microelectrode array optical inspection**

To enable optical inspection via light microscopy, an additional device, fabricated identically to the one shown in Figure 2, was aged under the same conditions. This surrogate device was used because the configuration of the accelerated aging setup, which includes additional components for electrochemical monitoring, obstructs direct optical access to the device undergoing periodic characterization.


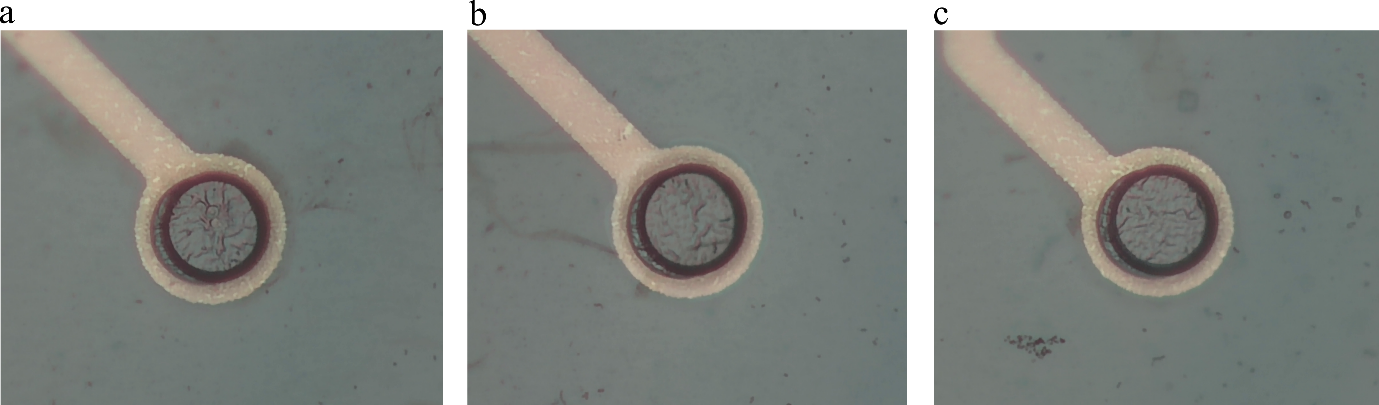
**Figure S7**. Optical inspection of microelectrodes. (a-c) Optical images of electrodes with same technology as in Figure 2 after 151 days of soaking in PBS at 57^o^C.

**Long term stimulation**

For completeness of the long-term stimulation study, we include here the data corresponding to stimulation with 1 billion biphasic current pulses of 100 μs pulse width, 100 μs interphase delay and 20 μA pulse amplitude. The amount of charge injected per phase is 0.4 mC/cm^2^ which is well above the clinical standards as described in the main text. The experiment with current pulses injection 0.4 mC/cm^2^ preceded those with 1 mC/cm^2^ , thus giving us confidence to raise the current levels since the data (see Figure S8) proved that there is not any major performance deterioration after the injection of 1 billion current pulses at a charge density of 0.4 mC/cm^2^ with the proposed technology of this study. Figure S8 compiles all the impedance data as well as the voltage response of the electrode to current pulses at the beginning and the end of the experiment.


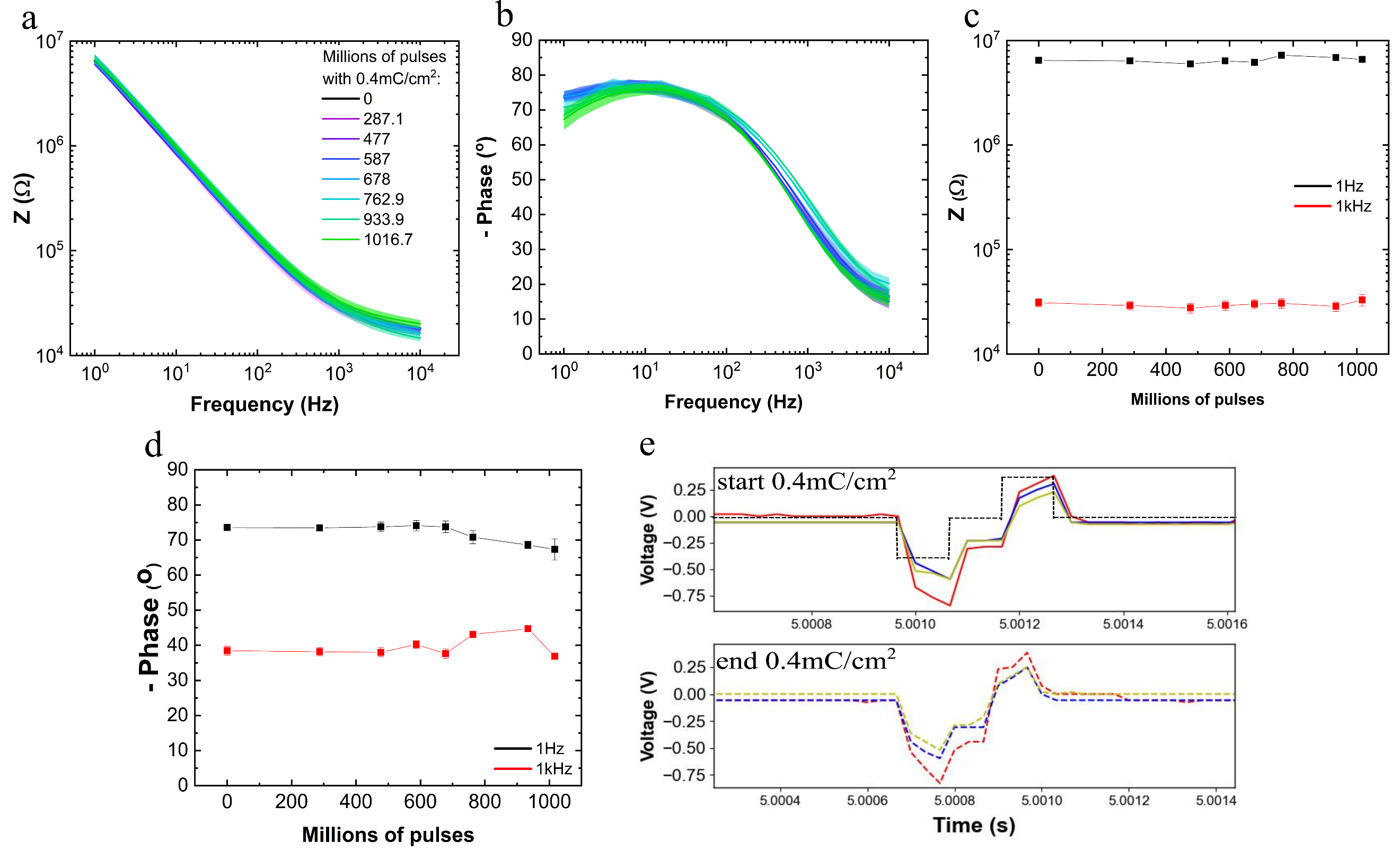


**Figure S8.** Assessment of rGO microelectrodes during accelerated stimulation at 0.4 mC/cm². (a-b) Mean impedance magnitude (a) and phase (b) versus frequency of rGO microelectrodes in PBS during the long-term simulation study with a charge injection of 0.4 mC/cm² (biphasic current pulses of 100 µs pulse width, 100 µs interphase delay, 50 µA pulse amplitude). (c-d) Mean (± standard error) impedance magnitude (c) and phase (d) at 1 Hz (black) and 1 kHz (red) versus the number of pulses (in millions). Data correspond to n=3 electrodes. (e) Electrode voltage polarization in response to the current stimulation pulses (solid lines: before stimulation study; dashed lines: after stimulation study) for n=3 electrodes (red, yellow, and blue). The black dashed line in the top plot represents the applied current pulse.

**Benchmarking of thin-film neurotechnologies: encapsulation and long-term studies**

| Substrate | Top encapsulation | Testing structures | Accelerated aging conditions | | Extrapolated lifetime | Reference |
| --- | --- | --- | --- | --- | --- | --- |
| Si/SiO2 | HfO2(100nm)/PDMS(50um) | IDEs | PBS, 23^o^C |  | | [24] |
| PI(5um) | PI(5um)/VPI Al_2_O_3_(20nm) | Mg tracks | PBS, 95 ^o^C | 1.3 years (25^o^C) | | [21] |
|  | PI(5um)/1 dyad |  |  | 0.4 years (25^o^C) | |  |
|  | PI(5um)/3 dyads |  |  | 2.6 years (25^o^C) | |  |
|  | PI(5um)/3 dyads/VPI Al_2_O_3_(20nm) |  |  | 4.7 years (25^o^C) | |  |
|  | PI(5um)/3 dyads/2x VPI Al_2_O_3_(20nm) |  |  | 5.8 years (25^o^C) | |  |
|  | PI(5um)/6 dyads |  |  | 6.4 years (25^o^C) | |  |
| Glass | Al_2_O_3_(52nm)/ParC(6um) | IDEs | PBS, 37 ^o^C | 260 days | | [22] |
|  |  |  | PBS, 57 ^o^C | 2.84 years (37 ^o^C) | |  |
|  |  |  | PBS, 67 ^o^C | 5.69 years (37 ^o^C) | |  |
|  |  |  | PBS, 80 ^o^C | 9.86 years (37 ^o^C) | |  |
| Glass | PI(5um)/HfO_2_(8nm)/Al_2_O_3_(20nm)/ HfO_2_(8nm)/ PI(5um) | Cu plane | PBS, 60 ^o^C | 14.3 years (37 ^o^C) | | [20] |
| PI(7.5um)/Al_2_O_3_(50nm) | Al_2_O_3_(50nm)/PI(2.5um) | IDEs | PBS, 57 ^o^C | 6.6 years (37 ^o^C) | | **this work** |

**Table S1**. Comparison of the encapsulation proposed in this paper with previous works

| Electrode material | Encapsulation | Elec. area (cm^2^) | Charge injection density (mC/cm^2^) | Number of current pulses | Stability | | Ref. |
| --- | --- | --- | --- | --- | --- | --- | --- |
|  |  |  |  |  | In vivo | In vitro |  |
| PEDOT:PSS | PI | 2.462 x 10^-3^ | 0.203 | 4.6 x 10^9^ | no | 386 days | [46] |
| PEDOT:PSS | PI/ PEDOT:PSS | 3.142 x 10^-4^ | 0.032 | 1.452 x 10^9^ | no | 84 days (70^o^C) | [33] |
| IrOx | PI | 4.524 x 10^-6^ | 0.055 | 1.9 x 10^6^ | 308 days | no | [32] |
| IrOx | PI | 2.4 x 10^-6^ | 1 | 1 x 10^9^ | 5 months | no | [7] |
| Pt-PDMS | PDMS | 1.43 x 10^-2^ | 0.042 | 1 x 10^9^ | 6 weeks | 110 days (37^o^C) | [12] |
| GC | PI | 7.07 x 10^-4^ | 0.43 | 5 x 10^6^ | no | no | [45] |
| GC/PEDOT:PSS | PI | 2.827 x 10^-5^ | 10.6 | 1 x 10^6^ | no | no |  |
| EGNITE | PI | 4.91 x 10^-6^ | 3 | 15 x 10^6^ | 90 days | no | [3] |
| EGNITE | PI | 4.91 x 10^-6^ | 1 | 1 x 10^8^ | 3 weeks | no | [8] |
| EGNITE | PI/Al_2_O_3_ | 4.91 x 10^-6^ | 1 | 1 x 10^9^ | no | 377 days (57^o^C) | **this work** |

**Table S2.** Comparison of various studies presenting thin-film electrodes designed for neuromodulation applications. The table provides details on the electrode material and encapsulation, electrode area (in cm²), the number of delivered current pulses and charge injected per phase (in mC/cm^2^), the long-term stability of the electrode under in vivo and in vitro conditions and corresponding references. 'GC' refers to Glassy Carbon, while 'EGNITE' denotes the nanoporous graphene electrodes investigated in this work.
